# Preparation, Physicochemical Characterization and Biological Evaluation of some Hesperidin Metal Complexes 

**Published:** 2014

**Authors:** Safa Daoud, Fatma U Afifi, Amal G Al-Bakri, Violet Kasabri, Imad I Hamdan

**Affiliations:** a *Department of Pharmaceutical Sciences*, *Faculty of Pharmacy, University of Jordan, Amman, 11942, Jordan. *; b *Department of Pharmaceutics and Pharmaceutical Technology*, *Faculty of Pharmacy, University of Jordan, Amman, 11942, Jordan.*; c *Department of Biopharmaceutics and Clinical Pharmacy*, *Faculty of Pharmacy, University of Jordan, Amman, 11942, Jordan. *

**Keywords:** Hesperidin, Chelation of polyphenols, Metals, Biological activity

## Abstract

The ability of hesperidin (HP) to form complexes with five metals; cobalt, nickel, zinc, calcium and magnesium was investigated. The complexation was studied using U.V spectroscopic titration, in methanol as well as aqueous buffer solutions (physiological conditions). Potential complexes were studied by IR and NMR spectroscopy, melting point and their solubility were also evaluated. The interaction of HP and its metal complexes with DNA was investigated by U.V spectroscopy. HP and its potential complexes were also tested for their ability to inhibit alpha amylase and alpha glucosidase enzymes. The results indicated that HP can form 1:1 complexes with cobalt, nickel and zinc in methanolic solution but not in aqueous buffers. Both HP and its metal complexes were found to intercalate DNA, at physiological condition, with preference to GC rich sequences. HP-metal complexes appeared to have higher affinity towards poly A DNA than the free HP. Neither HP nor its complexes exhibited antimicrobial activity against Staphylococcus aureus, Escherichia coli, Pseudomonas aeruginosa or Candida albicans. Results showed that HP has little inhibitory action on glucosidase and amylase enzymes with no obvious effect of complexation on the behavior of free HP.

In conclusion HP was shown to form 1:complexes with the studied metal in methanol but not in aqueous buffer solutions. In presence of DNA however, complex formation in aqueous solutions seem to be encouraged with differential effect between the complexes and free HP.

## Introduction

**Figure 1:**
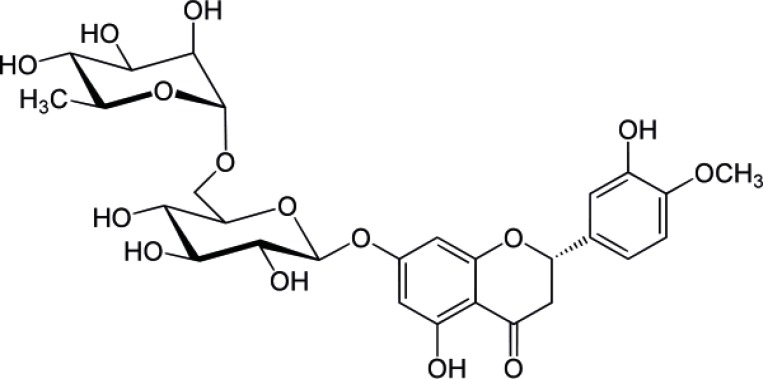
Structure of Hesperidin

Hesperidin (HP) is a flavonoid (Figure 1) that is abundantly found in citrus fruits. HP which consists of a glycone (hesperitin) attached to a disaccharide unit composed of rhamnose and glucose is found mainly in the peel of orange and lemon and usually isolated from *Citrus aurantium*, *C. sinensis *(Rutaceae). HP has been shown to inhibit skin carcinogenesis. In a relatively recent study HP was shown to have cytotoxic effect in colon cancer. Although the mechanism of cytotoxic action of HP is not well understood; it was shown that, in colon cancer cell apoptosis is achieved through activation of the enzyme caspase. The free aglycone (hesperetin) was also shown to have cytotoxic activity against different human cancer cell lines (1-4). In addition to the specific cytotoxic activity of HP, it was shown to have antioxidant activity which is a property shared by many other flavonoids (5). HP exhibits a significant effect on various metabolic pathways leading to hypoglycemia and hypocholesterolemia. HP and its aglycon were demonstrated to have modest alpha amylase inhibitory activities which might help explain their hypoglycemic effect. Furthermore, HP can also prevent microvascular leakage by virtue of its vaso-protective action through the inhibition of the enzyme hyaluronidase which is reported to regulate the permeability of capillary walls and supporting tissue (6-10). 

In spite of the various beneficial effects of HP on the biological system, the bioavailability of HP in human subjects seems to be limited. This was generally explained as a result of limited absorbability as intact glycoside and the low aqueous solubility of HP. Reasonable concentrations of HP were detectable (2.2 μ mol/L) in plasma after consumption of orange juice. Nevertheless, epidemiologic studies exploring the role of flavonoids in human health have been inconclusive. Some studies support a protective effect of flavonoid consumption in cardiovascular diseases and cancer, other studies demonstrate no effect, and a few studies suggest even potential harm (11-14). 

Drug metal complexation is a well known phenomenon that has gained increasing interest in recent years (15). Complexes of certain drugs may improve their physicochemical and pharmacological properties or in some cases decrease their potential side effects (16). Flavonoids and more generally phenolic compounds are known to form chelate complexes with various metal ions (17-19). 

In spite of the general expectations of the ability of HP, as a flavonoid, to form complexes with different metal ions, no particular studies have examined this possibility especially at physiological conditions. In particular, complexes with biologically relevant metals such as calcium, magnesium and zinc have not been reported for HP. Therefore, the objective of this work was to investigate the ability of HP to form complexes with selected metal ions (cobalt, zinc, calcium, magnesium, nickel) in physiologically relevant media. If the formation of metal-HP complexes was proven in physiological media, then it can be suggested that complexation might play an important role in the mechanism of action of the many reported biological activities of HP. 

## Experimental

*General*

MeOH (Lobachemie, India). Nickel (II) Chloride and Calcium Chloride, (Merck, Darmstadt). Zinc (II) Chloride, (Scharlau Chemie, Spain). Magnesium Sulphate dried, (Vickers Laboratories Ltd, England). Cobalt Acetate, (Hayashi Pure Chemical Industries Ltd, Japan). HEPES, (Sigma Chemicals, USA). Starch, (Uni-Chem, North Carolina, USA). α- Amylase and α- glucosidase (BioChemika- Sigma-Aldrich, Switzerland). Glucose GOD-PAP kit, (BioLabo Reagents, France). 

Microorganisms: *S. aureus *ATCC 6538P, *E. coli *ATCC 8739, *P. aeruginosa *ATCC 9027, *C. albicans *ATCC 10231 (all from OXOID, UK). Poly adenylic-cytiylic-guanylic acid-K salt and Poly adenylic acid- dodecathymidylic acid-Na salt (Sigma, Germany). All UV measurements were made by Spectroscan 80D UV-Vis spectrophotometer, Biotech. For alpha amylase test; Universal microplate reader, Bio-Tek instruments, ELx 800 UV (USA) was employed.

*Extraction and purification of HP*

240 gm of the dried and coarsely powdered orange peels were subjected to soxhlet extraction with 2 L petroleum ether for 1 h at 60 °C. The extract was thrown away and the powder dried (overnight, fume cabinet at room temperature ~24 ^o^C) and extracted again using 2 L of MeOH for further 2 h. The MeOH extract was concentrated until a syrupy residue obtained. The residue was dissolved using 6% acetic acid and left overnight to allow for precipitation and the precipitate was filtered, and dried (overnight, fume cabinet at room temperature ~24 ^o^C). Crude HP has been dissolved in dimethyl sulfoxide (DMSO) at 5 g/100 mL concentration using magnetic stirrer/hot plate where temperature was fixed at 60-80 °C until clear solution is obtained. Equivalent amount of DMSO was slowly added and the mixture kept at room temperature overnight for crystallization. The resulted crystals were filtered and washed with hot water then with butanol. Crystals were dried in a desiccator (3 days) and used for identification (20). No change in color, UV spectra, TLC performance was noticed for the material over a period of three months.

*Preparation of HP- metal complexes*

HP-metal complexes were prepared by dissolving 1.637 ×10^-4^ mole of HP (0.1 gm) in 250 mL of MeOH, equimolar amounts of the metal (*i.e*. 21.3, 22.3, 18.2, 19.7, 29.0 mg of either nickel chloride, zinc chloride, calcium chloride, magnesium sulphate, or cobalt acetate) were weighted and dissolved in 10 mL MeOH. The two MeOH solutions added to each other and mixed. The solution was left in fume hood until MeOH was completely evaporated. The residues were collected and placed in a desiccator (48 h) for further drying.

*Spectrophotometric study of complexation*

The potential complexation reactions were studied in MeOH, water, phosphate buffers (KH_2_PO_4_) at pH 9, 7.4, and 2.5 and in a mixture of MeOH and buffer pH 7.4 (50:50 v/v). Spectrophotometric titration was done by recording the spectra of solutions containing different HP to metal ratios. The stock solution of HP was prepared at a concentration of 165×10 ^-6^ M (0.01 g/100 mL). Equimolar solutions of each metal (165×10^-6^ M) were prepared. Series of test tubes for each metal were prepared by adding 3 mL of HP stock solution into each of them together with increasing volumes of metal solution. Then the volumes were completed to 15 mL using the solvent (MeOH or buffer) and mixed properly using vortex mixer. For each tube a blank solution was prepared which contained the same amount of metal without HP. The absorption spectrum for each solution was recorded against the corresponding blank in the range of 200-350 nm. Spectrophotometric titration was repeated three times for each metal- media combinations. 

*Determination of the solubility of HP and its complexes*

A stock solution of HP in MeOH was prepared at a concentration equal 10 mg% (1.64×10^-4^ M), from which four different dilutions were made. The absorbance values of these solutions were measured at the isosbestic point (290 nm); where both HP and complexes had the same molar absorptivity and plot against concentration, to construct calibration curves. For solubility in MeOH; 5 -10 mg of HP or its complexes were placed in separate test tubes, and 2 mL of MeOH were added to each sample. Samples were left in a shaker overnight at room temperature. Saturation was ensured in all solutions as evident by appearance of precipitate. Solutions were centrifuged, 0.5 mL of the supernatant of each sample was completed to 25 mL with MeOH, vortex mixed and the absorbance measured at 290 nm against the solvent. For solubility in phosphate buffer (20 mM, pH 7.4); the procedure was generally similar to above but 1 mL of supernatants was taken and evaporated under fume hood then the residues were dissolved in 2 mL MeOH and the absorption values of these solutions were recorded against blank buffer solution at isosbestic point.

*Evaluation of DNA binding properties of HP and its metal complexes*

HP and all its complexes were prepared as 40 μg/mL in (10:90) DMSO: HEPES-NaCl buffer solution (5 mM HEPES, 50 mM NaCl, pH=7.35). Two types of DNA were used throughout the study; poly adenylic-cytiylic-guanylic acid (poly ACG) and poly adenylic acid- dodecathymidylic acid (poly AT). The DNA concentrations were determined spectrophotometrically using molar absorptivities at 260 nm (14800 M^-1^ cm^-1^ and 12000 M^-1^ cm^-1^ for poly ACG and poly AT respectively). The absorption of DNA samples was measured at 280 nm for both types of DNA, and the ratio of absorption at 260/280 was used to indicate that they were sufficiently free from protein. Spectrophotometric titrations were performed for HP and complexes. Initially, 300 μL of tested sample and solvent were placed in sample and reference cuvettes, respectively. The first spectrum recorded then 10 μL of DNA solution was added each time, the solutions were allowed to incubate for 10 minutes before the absorption spectra were recorded. Similar increments of DNA were also added to blank cell at each time of addition to eliminate its effect on absorbance (4). 

*Antimicrobial assessment of HP and its metal complexes*

Antimicrobial activity of HP and its complexes in addition to the two metals of cobalt and nickel was assessed against *S. aureus*, *E. coli*, *P. aeruginosa *and *C. albicans *using the broth microdilution method according to CLSI (2005). HP and its complexes were prepared as stock solutions in DMSO at concentrations equal to 4 mg/mL. The broth microdilution method was performed in 96-well microtiter plate in which the first well was filled with (100 μL) double strength Mueller Hinton broth in case of bacteria and Sabouraud broth in case of *C. albicans*, while a single strength media was used to fill the rest of the wells. A volume of 100 μL of the tested preparation was added to the first well and subsequently series of two fold dilution were carried out across the plate with changing the tip of micropipette at each dilution step. Then 10 μL of overnight microbial culture standardized against McFarland standard was added to each well so as to obtain a final inoculum size of *ca*.1×10^6^ cfu/mL. Microtiter plates were incubated at 37 °C for 24 h, after which the microorganism growth was detected as turbidity relative to negative and positive controls. DMSO was used as positive control. Quality control antimicrobials of ampicillin and miconazole were used. MICs were expressed as the average of two successive concentration of the tested compound showing growth and no growth, respectively.

*In-vitro α amylase and α-glucosidase inhibitory activity assessment of HP and its complexes*

HP and its metal complexes were dissolved in water: DMSO (9:1) at a concentration of 0.5 mg/mL. Acarbose (which was used as a standard) was dissolved in the same solvent at a concentration of 0.5 and 1 mg/mL. The procedure for assessment of α- amylase and α- glucosidase inhibitory activity was adapted from (21) with some modifications. The effects of acarbose (standard) 500 and 1000 μg/mL were evaluated.

*Results and Discussion*

HP extracted from orange peel was obtained as a pale yellow powder (yield = 0.4 %) with melting point 260-263 °C which accords with reported values (22-23). The obtained UV, IR, and NMR spectroscopic data were identical to those previously reported for HP (22-23). Consequently, HP was positively identified by melting point, IR and NMR spectroscopy. 

**Figure 2:**
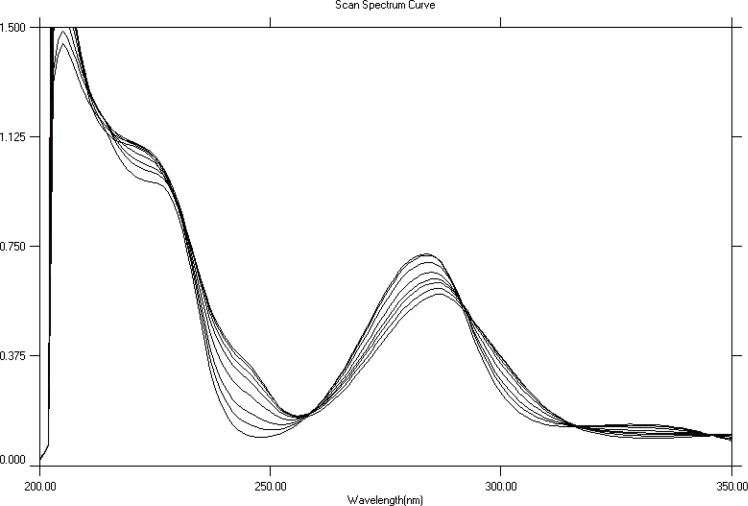
Absorption spectra of HP in methanol in absence (first spectrum from above) and in presence of increasing (from top to bottom) amounts of cobalt

HP metal complexation (in solution) was studied in different solvents using UV- visible spectrophotometer. These solvents included MeOH, water, phosphate buffer pH 9, phosphate buffer pH 7.4, phosphate buffer pH 2.5 and a mixture of methanol and buffer at pH 7.4 (50:50 v/v). HP spectrum showed obvious spectral changes when titrated with cobalt, nickel or zinc in MeOH. The maximum absorption band of HP which occurs at 284 nm showed a progressive and significant decrease, while a band centered at 300 nm increased with the addition of more metal. Titration spectra for each of the three metals (Co^+2^, Ni^+2^ and Zn^+2^) showed three isosbestic points (315 nm, 290 nm and 255 nm). Typical overlaid UV spectra for the titration of HP with cobalt are shown in Figure 2. The complexation of HP with Co^+2^, Ni^+2^, and Zn^+2^ is accompanied with a shift in λ_max _equal to 4 nm, 3 nm, and 1.5 nm respectively. 

However, no spectral changes were obtained where solvents other than MeOH were used. The absorption spectra of HP in MeOH solution titrated with calcium or magnesium also showed no changes. So, one can conclude that coordinate complexes can form between HP and studied metals in methanol (except with calcium and magnesium) but not in aqueous systems. 

**Figure 3:**
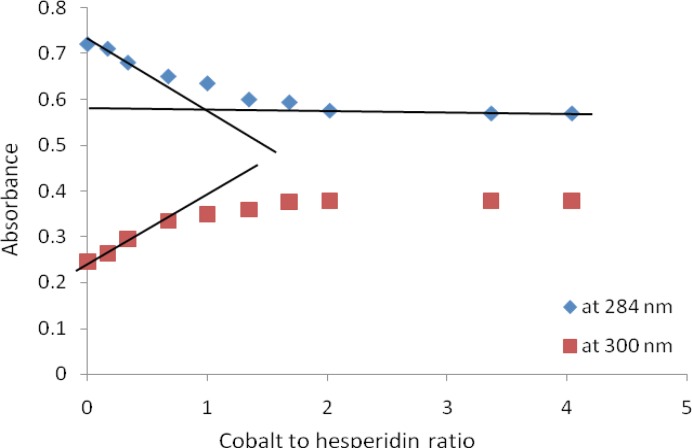
Absorbance versus molar ratio plot for the titration of HP with Co^+2^ . The solid lines show the extrapolation of the curves so that the intersection point represents the stoichiometric ratio of the formed complex

In order to determine the stoichiometry of complexes; the absorbance at 284 nm (λ _max_ of HP) and at 300 nm were plotted against molar ratios of metal to HP. These plots, (molar ratio plots) showed inflection points at ratio of metal: HP = 1 for all complexes which indicated a stoichiometry of 1:1 metal: HP. The molar ratio plot for titration of HP with Co^2+^ is shown in Figure 3. Formation constants (K_f_) of HP metal complexes were also estimated from U.V titration experiment (in MeOH) according to a previously reported method (24). These were: 4.88×10^5^ M^-1^, 13.99 ×10^5^ M^-1^ and 62.21 ×10^5^ M^-1^ for HP-Co^+2^, HP-Ni^+2^ and HP-Zn^+2^ complexes respectively.

Based on the structure of HP and previous studies with flavonoids, the coordination of HP with metal ions occurs most probably through carbonyl group at position 4 and the aromatic hydroxyl group at position 5 (4, 25). However, for strong complex formation to take place the aromatic hydroxyl should acquire a negative charge (ionized). Since the expected pKa value for the phenolic hydroxyl is around 10 then the pH of the solution must be above 10 to allow for significant ionization to take place. Thus, it could be concluded that HP did not form complexes with metal ions in aqueous systems at the studied range of pH values (2.^5^-^9^). 

In order to further investigate potential complex formation using other analytical techniques, attempts were made to obtain potential complexes in solid state (experimental section). **Table 1** represents the values of melting points, for the potential complexes, as average of three successive measurements. The observed significantly higher melting point for HP as compared to that of potential complexes, suggest formation of complexes between HP and each of cobalt, zinc, nickel and calcium. HP-Mg^+2^ showed no significant change (from that of HP) in melting point indicating no complex formation took place between HP and magnesium. The results of IR spectra did not provide any evidence for complex formation since no changes were observed in the IR spectra of the prepared complexes. 

NMR spectra did not give a clear evidence for complex formation, since no obvious changes were detected in HP-Co^+2^, HP-Zn^+2^, HP-Ca^+2^ and HP-Mg^+2^ potential complexes. However, the spectrum of the potential HP-Ni^+2^ complex, showed general broadness in most of the peaks indicating non-specific association between nickel and HP. The NMR spectra of complexes did not agree with our expectations of coordination between metal and HP through carbonyl group on C-4 involving the hydrogen of the hydroxyl on C-5, which would have led to major change in the chemical shift of this proton (OH-5).

A potential explanation for the observed IR and NMR results (*i.e*. lack of evidence on complexation), is that the employed method for the preparation of complexes in solid state was not efficient. In fact, most of the reported methods for the preparation of the flavonoid complexes with metals, adopted harder conditions *i.e. *refluxing the metal with the flavonoid for relatively long time (4). The employed method in the present study was softer than the reported methods, because a major aim of this study was to assess whether complex are really formed or not, between HP and metals under physiological or near physiological conditions.

A calibration curve was constructed for HP or its complexes by measuring absorbance at the isosbestic point (290 nm). The obtained calibration equation could be given by :

Y = 0.192x – 0.009 (R^2^ = 0.999), where x is the concentration in molar. The same equation was used for the determination of both of HP and its complexes because they have the same molar absorptiviy at isosbestic point. The obtained molar concentrations were converted to mg/mL unit. Table 2 summarizes the overall solubility data. In general HP and its potential metal complexes were significantly more soluble (~40 times) in methanol than in phosphate buffer (pH 7.4). No notable differences in solubility between HP or any of its complexes was observed when phosphate buffer pH 7.4 was employed as a solvent. This is consistent with the finding that complex formation did not take place in aqueous system. Therefore, HP existed only in free form in phosphate buffer and consequently no significant change in solubility was observed. 

On the other hand, there were obvious differences in solubility of HP and its potential complexes when MeOH was employed as a solvent. This observation reinforces the finding that complexation took place in MeOH. Moreover, HP-Ca^+2^ and HP-Mg^+2^ exhibited solubilities very close to that of free HP, which supports the findings that they did not really form complexes with HP. For other metals (Co^+2^, Ni^+2^ and Zn^+2^) their potential complexes exhibited slightly lower solubility than the free HP. 

**Figure 4:**
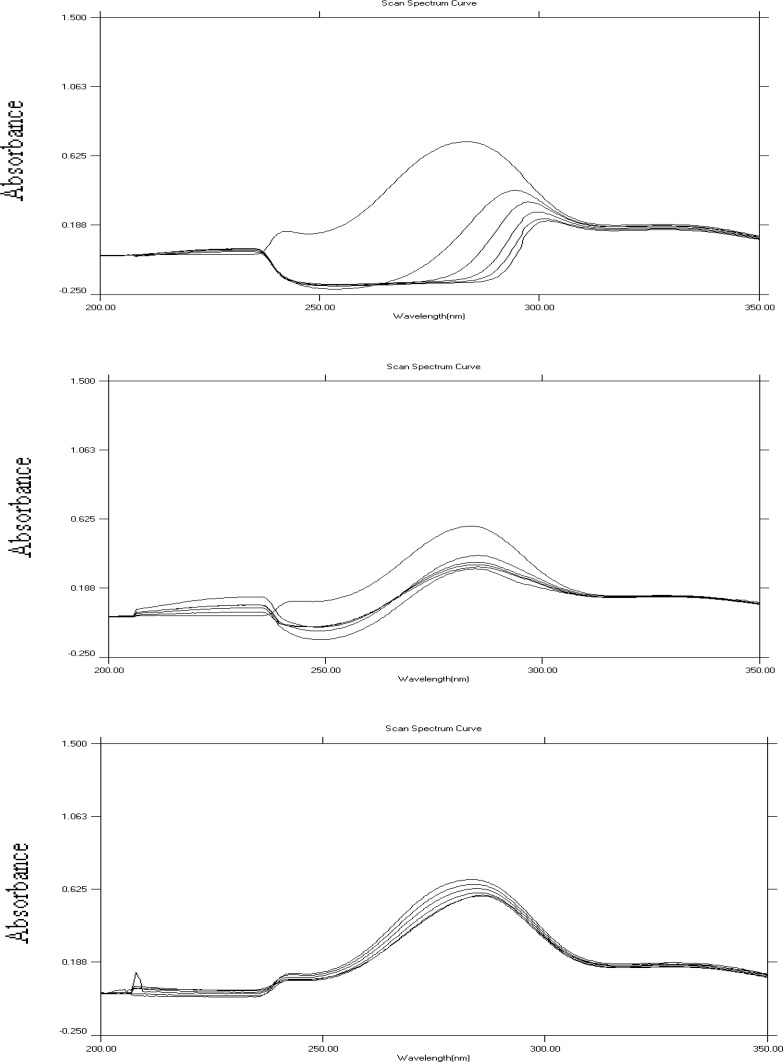
Absorption spectra of HP (A and C) or HP-Co^+2^ complex (B) in the absence and presence of increasing (from top to bottom, 0-50 μL DNA) amounts of poly ACG DNA (in A and B) and of poly A DNA (in C).

DNA plays a key role in cell replication and protein synthesis making it a good target for many drugs including antiviral, antibacterial and anticancer drugs (19). There have been some evidences on the interaction of some flavonoids with calf thymus DNA (26). Binding of HP and its metal complexes to DNA was evaluated using absorption titration. Spectroscopic titrations were carried out for HP and all potential complexes using two types of DNA: poly A and poly ACG. Sample spectra obtained for the titration of HP, HP-Co^+2^ complexes with ploy A and poly ACG DNA are shown in Figure 4. **Table 3 **summarizes the results obtained for the titration of HP and its potential complexes with poly ACG and poly A DNA. It could be concluded that HP and its complexes interact with poly ACG in a stronger manner compared to that with poly A DNA as indicated by the severe hypochromic shifts observed in the spectroscopic titration with poly ACG DNA. The titration spectra with poly ACG DNA showed a considerable drop in absorption of HP (or complexes) at about 284 nm upon increasing the concentration of DNA. The observed hypochromisms were so large that the band at 284 nm nearly disappeared. The binding also resulted in significant bathochromic shifts. The obvious hypochromic and bathochromic shifts are characteristic features of intercalative mode of binding to DNA (27). It is noteworthy that, while HP and other complexes (except HP-Co^+2^) behaved similarly (in term of hypochromic and bathochromic shifts); HP-Co^+2^ complex exhibited obviously different behavior.

With poly A DNA titration, the little hypochromic shifts, may indicate electrostatic interaction or groove binding of HP and its complexes to DNA (27). The results showed higher percentages of hypochromic shift in presence of metals, indicating higher affinity of complexes than free HP towards poly A DNA which is in accordance with literature (19). Therefore, this experiment demonstrated that, metal ions (through formation of complexes) may modulate the affinity of intercalative binding to DNA. Moreover HP and its potential metal complexes appeared to selectively bind to GC (guanine– cytosine) rich DNA sequences as compared to poly A sequences. Selectivity of binding to DNA sequences is very important in the development of more potent cytotoxic (anticancer) agents with fewer side effects.

Interestingly, even magnesium and calcium (which were not shown to form complexes with HP) appeared to modify the binding of HP to DNA. This can be explained as the presence of the rather more lipophillic environment of base pairs which encourages the lipophillic complex to escape the hydrophilic surroundings and pushing the complexation equilibrium forward. These results have particular importance as calcium and magnesium are relatively abundant physiological ions in cells. 

The antimicrobial activity of HP and its metal complexes against *Staphylococcus aureus*, *Escherichia coli*, *Pseudomonas aeruginosa *and *Candida albicans *was evaluated. The results of antimicrobial testing showed that HP and all its complexes were not active against *S. aureus*, *E. coli *and *P. aeruginosa. *Two of the tested compounds; HP-Co^+2^ and HP-Ni^+2^, showed inhibition activity towards *C. albicans *with MIC equal to 187.5 μg/mL (n = 3, RSD = 0). So the activity of their corresponding metals; Co^+2^ and Ni^+2^, were tested alone. Ni^+2^ and Co^+2^ showed inhibitory activity towards *C. albicans *with MIC equal to 46.8 μg/mL (n = 3, RSD = 0). So, it could be concluded that the observed inhibition of *C. albicans *was due to transition metals themselves rather than to HP complexes.

Alpha amylase is a calcium dependent enzyme, so it is likely that the ability of a compound to form complexes with calcium may influence the α-amylase inhibitory activity. This is particularly important, since many of reported α-amylase inhibitors were good chelators such as tetracycline (28). Thus, it is reasonable to expect that HP and its potential complexes have the possibility to effectively suppress α-amylase and /or α-glucosidase activity and so they were tested as inhibitors for both enzymes. The obtained percentages of inhibition were: 100.1%, 9.08%, 8.6%, 3.5%, 9.8%, 12.1%, and 12.7% for acarbose, HP, HP-Co^+2^, HP-Zn^+^, HP-Ca^+2^, HP-Mg^+2^, HP-Ni^+2^, respectively. In general HP and its potential complexes were far less potent than the standard inhibitor acarbose. However, complexation did not produce any notable improvement on free HP behavior**. **Interestingly, the potential HP-metal complexes appeared to have different activities *i.e*. HP- Zn^+2^ exhibited the least inhibition activity (3.5%), where HP-Ni+2 was the most active one (12.7%). These differences however, did not correlate well with the estimated stability constants of complexes. 

## Conclusion

U.V studies indicated complex formation between HP and some metal ions (HP-Co^+2^, HP-Zn^+2^, HP-Ni^+2^) in MeOH. Under Physiological conditions (aqueous solvent and near neutral pH value) none of the tested metals appeared to form complexes with HP. IR and NMR studies did not provide evidence for complexation in solid state which could suggest that the employed method for the preparation of complexes in solid state might not be effective. HP and its metal complexes were found to bind (intercalate) to DNA, at physiological condition, with obvious selectivity to sequences that are rich in GC bases compared to adenine bases. The presence of metal ions was found to increase the affinity of intercalative binding to DNA, which suggests that under the non polar local environment of DNA base pairs, HP is encouraged to form complex with metals ions.

Although HP and its potential complexes could bind to DNA efficiently, they did not produce any inhibitory activity against *S. aureus, E. coli*, *P. aeruginosa *and *C. albicans*. A possible explanation for this result could be the inability of HP and its complexes to enter inside the cells. HP has little inhibitory action against alpha amylase and glucosidase enzymes and complexation did not produce significant improvements on free HP behavior.
